# Endarterectomy treatment for patients suffering from a new episode of pulmonary embolism, after screening for chronic thromboembolic pulmonary hypertension

**DOI:** 10.1186/s13054-022-04037-6

**Published:** 2022-06-07

**Authors:** Patrick M. Honore, Sebastien Redant, Pharan Djimafo, Sydney Blackman, Ibrahim Bousbiat, Thierry Preseau, Bogdan Vasile Cismas, Keitiane Kaefer, Leonel Barreto Gutierrez, Sami Anane, Andrea Gallerani, Rachid Attou

**Affiliations:** 1 ICU Department, Centre Hospitalier Universitaire Brugmann-Brugmann University Hospital, Place Van Gehuchtenplein, 4, 1020 Brussels, Belgium; 2 ULB University, Brussels, Belgium; 3 ED Department, Centre Hospitalier Universitaire Brugmann, Brussels, Belgium

“Advanced management of intermediate-high risk pulmonary embolism (PE)” shows that there is a higher mortality rate and recurrence of PE in patients treated by repeat-dose thrombolysis compared to surgical embolectomy [1]. The study also indicates that all bleeding events in repeated thrombolytic therapy were fatal [1]. In addition, among the 174,322 patients hospitalized for PE in the New York State Registry, there was no short-term difference in mortality between these two treatments [1]. Moreover, patients who underwent surgical pulmonary embolectomy had lower rates of stroke, recurrent PE, and need for re-intervention [1]. Due to the fatal outcomes, patients who have developed chronic thromboembolic pulmonary hypertension (CTEPH) after an initial PE should not receive a second thrombolytic therapy [2]. Taking the high recurrence rates in patients with CTEPH into consideration, CTEPH should be diagnosed beforehand, and could be treated by pulmonary endarterectomy (PEA), a challenging procedure with complex patient selection and perioperative management requiring significant expertise [2, 3]. Nevertheless, if treated in time, patients can be cured, even though they may need temporarily veno-arterial or veno-venous extracorporeal membrane oxygenation (VA or VV-ECMO) [2]. Patients suffering from a PE recurrence after embolectomy should not undergo a new embolectomy treatment because of the high fatality rate, and instead should be screened quickly for CTEPH and undergo a PEA as treatment and PE prevention [2]. The management of distal thromboembolic lesions with PEA remains a challenge. Limited distal CTEPH is not considered an absolute contraindication for a PEA, with up to 50% of patients having incomplete thrombi removal or extensive pulmonary microvascular disease. Medical therapies that target the underlying pulmonary microvascular disease can offer symptomatic and hemodynamic benefits, although they do not deal with the core mechanism of the disease which require the removal of thromboembolic material from pulmonary vasculature. Recent research has provided evidence suggesting balloon pulmonary angioplasty (BPA) is one treatment option for inoperable CTEPH and recurrent/persistent pulmonary hypertension after PEA [4]. Thrombolysis and surgical embolectomy may not be the cure for everyone, especially in case of repeated PEs [3, 4]. A clear step-by-step approach needs to be implemented in case of failed thrombolysis. In the case of CTEPH, diagnosis should not be overlooked, as PEA and BPA might be lifesaving [2].

## Data Availability

Not applicable.

## Abbreviations

PE: Pulmonary embolism
CTEPH: Chronic thromboembolic pulmonary hypertension
PEA: Pulmonary endarterectomy
BPA: Balloon pulmonary angioplasty
VA-ECMO: Veno-arterial extracorporeal membrane oxygenation
VV-ECMO: Veno-venous extracorporeal membrane oxygenation
